# LRG1 expression indicates unfavorable clinical outcome in hepatocellular carcinoma

**DOI:** 10.18632/oncotarget.5967

**Published:** 2015-10-19

**Authors:** Chun-Hua Wang, Min Li, Li-Li Liu, Ruo-Yao Zhou, Jia Fu, Chris Zhiyi Zhang, Jing-Ping Yun

**Affiliations:** ^1^ Sun Yat-sen University Cancer Center, State Key Laboratory of Oncology in South China, Collaborative Innovation Center for Cancer Medicine, Guangzhou 510060, China; ^2^ Department of Pathology, Sun Yat-sen University Cancer Center, Guangzhou 510060, China; ^3^ Okemos High School, Okemos MI 48864, USA

**Keywords:** LRG1, prognosis, immunohistochemistry, hepatocellular carcinoma

## Abstract

Leucine-rich-alpha-2-glycoprotein1 (LRG1) is a novel oncogene-associated protein which has been clarified vital to the progression of human cancers, but its role in hepatocellular carcinoma (HCC) remains unclear. Here, we showed that the expression of LRG1 was noticeably increased in HCC tissues, compared to the nontumorous tissues. High LRG1 expression was significantly associated with tumor size (*P* = 0.004), tumor differentiation (*P* = 0.010), TNM stage (*P* < 0.001) and vascular invasion (*P* = 0.019). Kaplan-Meier analysis showed that LRG1 expression was closely correlated to overall survival and disease-free survival in a training cohort of 474 patients with HCC. The correlation was further validated in an independent cohort of 303 HCC patients. The prognostic implication of LRG1 was confirmed by stratified survival analyses. Multivariate Cox regression model indicated LRG1 as an independent poor prognostic indicator for overall survival (Hazard ratio = 1.582, 95% confident interval: 1.345–1.862, *P* < 0.001) and disease-free survival (Hazard ratio = 1.280, 95% confident interval: 1.037–1.581, *P* = 0.022) in HCC. *In vitro* data showed that LRG1 markedly promoted cell migration but has no effect on cell proliferation. Collectively, our data show that LRG1 is markedly up-regulated and serves as an independent factor of poor outcomes in HCC. Our study therefore provides a promising biomarker for prognostic prediction in clinical management of HCC.

## INTRODUCTION

Hepatocellular carcinoma (HCC) represents the fifth most common cancer, and is the third leading cause of cancer death worldwide [1]. Although progress has been made in the clinical treatment of HCC, patients still suffer poor prognosis because of intrahepatic metastases or postsurgical recurrence [2]. Many factors are attributed to the increase the risk of HCC preneoplastic liver lesions, including hepatitis virus (HBV or HCV) infection, alcohol abuse and aflatoxin exposure [3]. To date, the molecular mechanisms of HCC development and progression remain obscure. It is very helpful to identify risk factors and biomarkers for early diagnosis and prognostic prediction in patients with HCC.

Leucine-rich-2-glycoprotein (LRG) was first identified as a trace protein in human serum [4]. Leucine-rich-alpha-2-glycoprotein1 (LRG1), a membrane-associated LRR family member, has been predicted as a regulator of glucan synthesis [5], cell adhesion [6], and cell migration [7]. LRG1 has also been proposed to play a role in cell survival and apoptosis [8, 9]. Wang et al. showed that LRG1 was capable of accelerate angiogenesis by directly binding to the TGF-β accessory receptor endoglin to activate Smad1/5/8 signaling pathway [10]. The role of LRG1 in malignant carcinomas has not been well studied. Increased LRG1 expression has been demonstrated in ovarian cancer [11], non-small cell lung cancer [12], gastric cancer [13], pancreatic cancer [14] and leukemia [15]. These data suggest a potential role of LRG1 in cancer progression.

LRG1 has been implicated in serum and/or plasma biomarker for diagnosis [16, 17]. Its expression level and prognostic value have been revealed in endometrial carcinoma [18]. In this study, we detected the expression of LRG1 at both mRNA and protein levels in HCC cell lines and tissues. The relationship between LRG1 and clinicopathological features of HCC patients was determined. The prognostic value of LRG1 was accessed in 777 archived paraffin-embedded HCC clinical samples.

## RESULTS

### Expression of LRG1 in HCC samples by qRT-PCR and western blot

**Figure 1:**
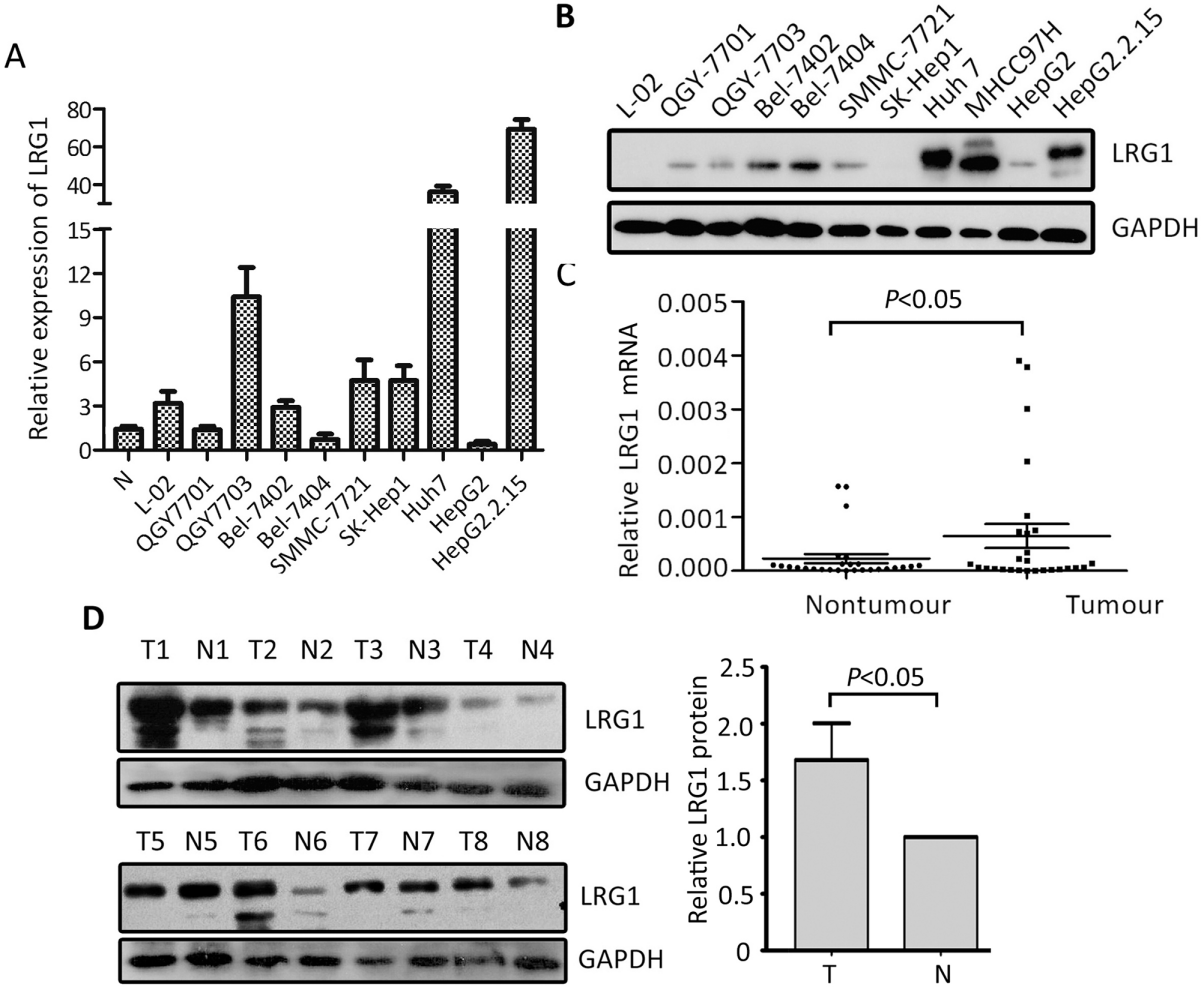
LRG1 expression is determined in HCC by qRT-PCR and western blot **A, B.** The expression of LRG1 at mRNA (A) and protein (B) levels in HCC cell lines was examined. **C.** The mRNA levels of LRG1 in HCC (T) and corresponding adjacent liver tissue (N) were determined in 27 patients. **D.** Expression of LRG1 protein in 32 paired HCC (T) and adjacent liver tissues (N) were examined by western blot. Representative images were shown.

The expression of LRG1 was determined in HCC cells lines by qRT-PCR and western blot. Results showed that LRG1 mRNA levels in most HCC cell lines was up-regulated, compared to adjacent nontumorous tissues and the immortalized hepatic cell (L-02) (Figure 1A). Consistently, the protein LRG1 protein levels were noticeably increased, compared to L-02 cell (Figure 1B). In 27 HCC fresh samples, LRG1 mRNA was significantly up-regulated in HCC tissues (Figure 1C), compared to the corresponding nontumorous tissues. The increase of LRG1 protein in 32 pairs of fresh HCC tissues was also observed (Figure 1D).

### Expression of LRG1 in HCC samples by immunohistochemistry

**Figure 2:**
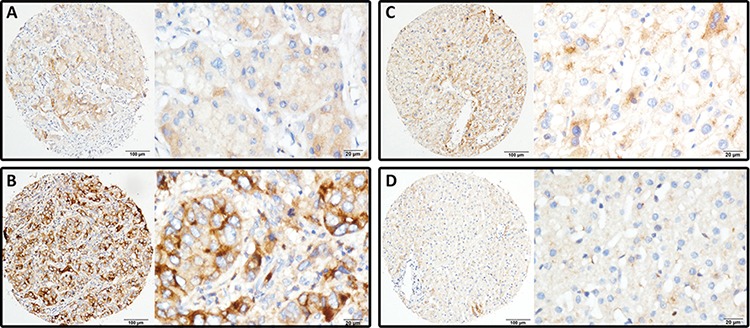
LRG1 expression is determined in HCC by immunohistochemistry LRG1 was presented predominantly in cytoplasm within tumor and normal liver cells. The micrographs showed weak **A.** and strong **B.** staining in HCC, along with positive **C.** and negative **D.** staining in nontumorous liver tissues (Left panel: magnification ×100; right panel: magnification ×400).

To further examine the expression of LRG1 in HCC tissues, 777 paraffin-embedded HCC samples were collected to construct TMA. As shown by the result of TMA-based IHC, immunoreactivities of LRG1 were mainly present in the cytoplasm in most of the cancer cells (Figure 2A & 2B). Positive expression of LRG1 was depicted in a few of HCC cases in nontumorous tissue (Figure 2C & 2D), but in 85.8% (667/777) of HCC tissues. Furthermore, in 67.7% (526/777) of the samples, LRG1 expression in HCC was found higher than that in nontumorous tissue.

### Association of LRG1 expression and clinical features in HCC

To determine the clinical significance of LRG1 in HCC, the relationship between expression of LRG1 and clinicopathological parameters was analyzed. Patients were randomly separated into training (*n* = 474) or validation (*n* = 303) cohort. According to the median of IHC score (4.63), high LRG1 expression was identified in 51.4% (399 777) of cases. In the training cohort, high LRG1 expression was more likely to present advanced clinical characters, including higher advanced clinical stage (*P* = 0.008), tumor size (*P* = 0.037) and worse tumor differentiation (*P* = 0.030). In the validation cohort, high LRG1 expression was frequently associated with higher advanced clinical stage (*P* = 0.012), worse tumor differentiation (*P* = 0.035) and vascular invasion (*P* = 0.006) (Supplementary Table S1). In the overall cohort, patients with high LRG1 expression was accompanied with higher advanced clinical stage (*P* = 0.010), larger tumor size (*P* = 0.004), more vascular invasion (*P* = 0.019) and worse tumor differentiation (*P* < 0.001) (Table 1).

**Table 1:** Correlation of clinicopathological parameters and LRG1 expression in overall cohort (*n* = 777)

Variable	Overall cohort			
	All cases	Low expression	High expression	*P* value^a^
Age (years)^b^				0.475
<49	368	184 (50.0%)	184 (50.0%)	
≥49	409	194 (47.4%)	215 (52.6%)	
Gender				0.092
Male	699	333 (47.6%)	366 (52.4%)	
Female	78	45 (57.7%)	33 (42.3%)	
HBsAg				0.913
Positive	663	322 (48.6%)	341 (51.4%)	
Negative	114	56 (49.1%)	58 (50.9%)	
AFP (ng/ml)				0.131
<20	229	121 (52.8%)	108 (47.2%)	
≥20	548	257 (46.9%)	291 (53.1%)	
Cirrhosis				0.386
Yes	619	306 (49.4%)	313 (50.6%)	
No	158	72 (45.6%)	86 (54.4%)	
Tumor size (cm)				**0.004**
<5	235	133 (56.6%)	102 (43.4%)	
≥5	542	245 (45.2%)	297 (54.8%)	
Tumor multiplicity				0.052
Single	464	239 (51.5%)	225 (48.5%)	
Multiple	313	139 (44.4%)	174 (55.6%)	
Differentiation				**0.010**
Well-Moderate	533	276 (51.8%)	257 (48.2%)	
Poor-undifferentiated	244	102 (41.8%)	142 (58.2%)	
TNM				**0.000**
I–II	392	216 (55.1%)	176 (44.9%)	
III–IV	385	162 (42.1%)	223 (57.9%)	
Vascular invasion				**0.019**
Yes	187	77 (41.2%)	110 (58.8%)	
No	590	301 (51.0%)	289 (49.0%)	
Involucrum				0.146
Complete	268	140 (52.2%)	128 (47.8%)	
Incomplete	509	238 (46.8%)	271 (53.2%)	

a Chi-square test

b Median age; AFP, alpha-fetoprotein; HBsAg, hepatitis B surface antigen.

### Association of LRG1 expression and clinical outcomes in HCC

**Figure 3:**
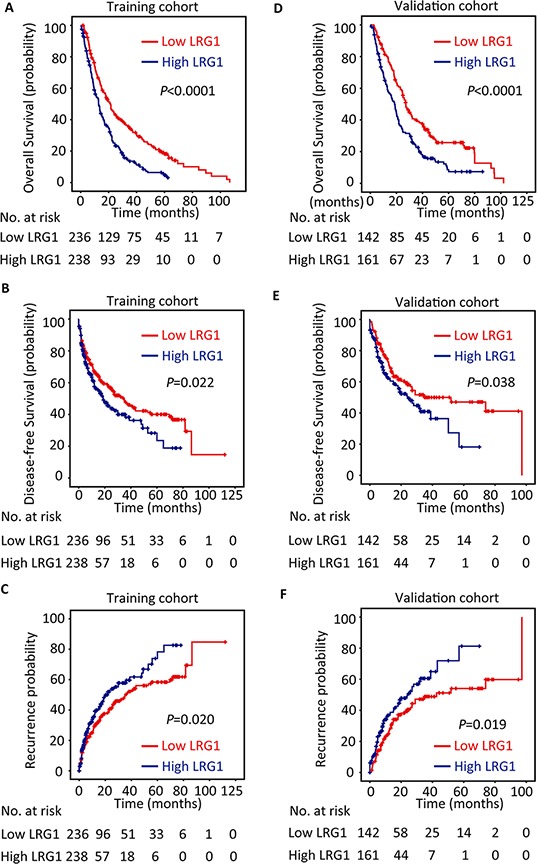
LRG1 expression is correlated with poor outcome in training and validation cohorts The patients with HCC in the training (*n* = 474) and validation (*n* = 303) cohorts were divided into high and low LRG1 expression groups according to median of IHC score. Kaplan-Meier analysis was conducted to disclose the relationship of LRG1 expression and the overall survival **A, B.** disease-free survival **C, D.** and recurrence probability **E, F.** (log-rank test).

To determine the prognostic impact of LRG1 on HCC patients, Kaplan–Meier survival analysis was conducted. Results revealed HCC cases with high LRG1 expression were often accompanied with significantly worse prognosis, in terms of overall survival (*P* < 0.001), disease-free survival (*P* = 0.022) and recurrence probability (*P* = 0.020) in the training cohort (log-rank test; Figure 3A–3C). This was validated in validation cohort by showing that increase of LRG1 was associated with inferior overall survival (*P* < 0.001), disease-free survival (*P* = 0.038) and higher tendency of recurrence (*P* = 0.019) (log-rank test; Figure 3D–3F).

**Figure 4:**
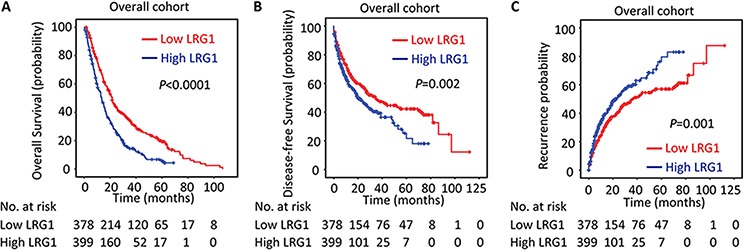
LRG1 expression is correlated with poor outcome in overall cohort The association of LRG1 expression and the overall survival **A.** disease-free survival **B.** and recurrence probability **C.** in the overall cohort (*n* = 777) was determined by Kaplan-Meier analysis (log-rank test).

**Figure 5:**
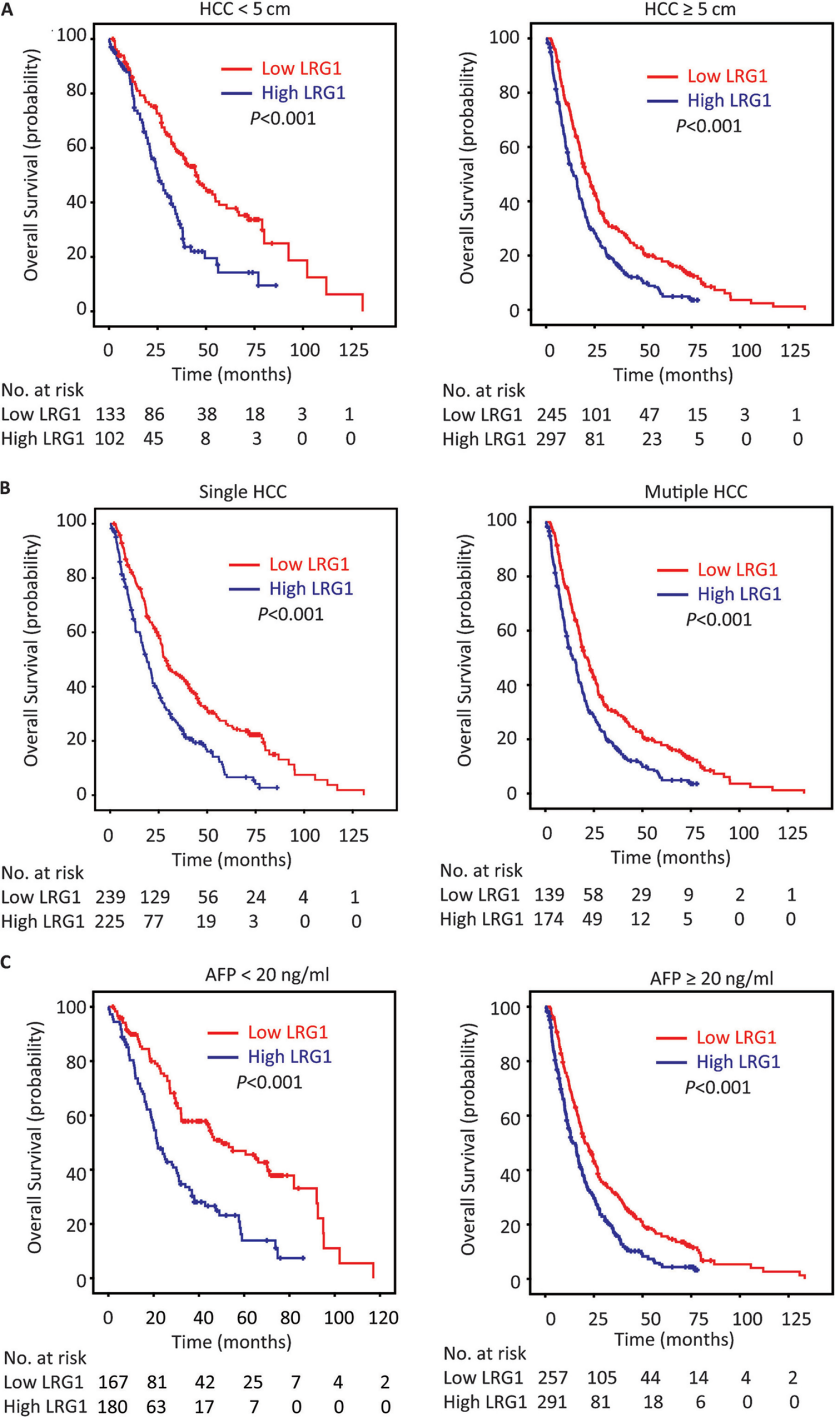
LRG1 expression is associated with overall survival in subgroups of HCC patients Stratified survival analyses showed that LRG1 expression was connected with overall survival in small and large HCC **A.** in unique and multinodular HCC **B.** and in HCC with low and high level of serum AFP **C.** (log-rank test).

In line with the results of the individual cohort, patients with high LRG1 expression were likely to have shorter overall survival (*P* < 0.001), disease-free survival (*P* = 0.002) and higher recurrence probability (*P* = 0.001) in the overall cohort (log-rank test; Figure 4). Stratified survival analyses further confirmed the prognostic significance of LRG1. Data showed that LRG1 expression was connected with overall survival in small and large HCC (Figure 5A), in single and multiple HCC (Figure 5B), in HCC with low and high level of serum AFP (Figure 5C), in HCC with negative and positive HBV infection (Supplementary Figure S1A), in HCC at I-II and III-IV TNM stage (Supplementary Figure S1B), and in HCC with well-moderate and poor-undifferentiated differentiation (Supplementary Figure S1C).

### Univariate and multivariate analyses of prognostic variables in HCC

To evaluate the representativeness of our samples, univariate analyses were performed. LRG1, as well as tumor size, serum AFP level, tumor multiplicity, clinical stage, vascular invasion, and tumor differentiation were shown to be responsible for the outcome of overall survival in both training cohort and validation cohort (Supplementary Table S2). Multivariate analyses were conducted to determine the independent prognostic value of LRG1. After adjusting for the prognostic factors established in the univariate analysis, data indicated that LRG1 expression was an independent prognostic factor for overall survival in both training cohort (hazard ratio (HR) = 1.699, 95% confident interval (CI): 1.383–2.087, *P* < 0.001) and validation cohort (HR = 1.421, 95% CI: 1.080–1.867, *P* = 0.011). The independence of LRG1 in predicting disease-free survival for patients in both cohorts was also investigated (Supplementary Table S3).

In the overall cohort of 777 patients with HCC, LRG1, along with tumor size, tumor multiplicity, serum level of AFP, tumor differentiation, vascular invasion and TNM, were identified as independent prognostic factors (Table 2). Multivariate analysis indicated LRG1 as an independent factor for overall survival (HR = 1.582, 95% CI: 1.345–1.862, *P* < 0.001) and disease-free survival (HR = 1.280, 95% CI: 1.037–1.581, *P* = 0.022) (Table 2).

**Table 2:** Univariate and multivariate analyses of clinicopathological and LRG1 expression for overall and disease-free survival in overall cohort (*n* = 777)

Variables	Univariate analysis		Multivariate analysis	
	HR (95% CI)	*P* value	HR (95% CI)	*P* value
**Overall survival**				
Age (<49 vs. ≥49 years)	1.034 (0.884–1.209)	0.679		
Gender (female vs. male)	0.928 (0.713–1.209)	0.581		
HBV (positive vs. negative)	0.991 (0.795–1.234)	0.935		
Tumor size (<5 vs. ≥5 cm)	1.934 (1.616–2.335)	**0.000**	1.620 (1.329–1.974)	**0.000**
Tumor multiplicity (single vs. multiple)	1.309 (1.118–1.533)	**0.001**	1.021 (0.838–1.245)	0.835
Invonucrum (absent vs. present)	1.123 (0.956–1.319)	0.159		
Liver cirrhosis (yes vs. no)	1.072 (0.876–1.312)	0.497		
AFP (<20 vs. ≥20 ng/mL)	1.932 (1.608–2.321)	**0.000**	1.722 (1.430–2.075)	**0.000**
Vascular invasion (yes vs. no)	1.901 (1.586–2.278)	**0.000**	1.454 (1.187–1.782)	**0.000**
Tumor differentiation	1.356 (1.147–1.603)	**0.000**	1.117 (0.941–1.327)	0.206
TNM (I–II vs. III–IV)	1.704 (1.455–1.995)	**0.000**	1.151 (0.919–1.440)	0.220
LRG1 expression (low vs. high)	1.756 (1.496–2.063)	**0.000**	1.582 (1.345–1.862)	**0.000**
**Disease-free survival**				
Age (<49 vs. ≥49 years)	1.046 (0.851–1.286)	0.669		
Gender (female vs. male)	1.123 (0.806–1.567)	0.493		
HBV (positive vs. negative)	1.098 (0.812–1.484)	0.544		
Tumor size (<5 vs. ≥5 cm)	1.570 (1.195–1.901)	**0.001**	1.356 (1.078–1.727)	**0.010**
Tumor multiplicity (single vs. multiple)	1.093 (0.886–1.349)	0.404		
Invonucrum (absent vs. present)	1.187 (0.960–1.468)	0.112		
Liver cirrhosis (yes vs. no)	1.076 (0.830–1.395)	0.581		
AFP (<20 vs. ≥20 ng/mL)	1.601 (1.267–2.024)	**0.000**	1.488 (1.175–1.885)	**0.001**
Vascular invasion (yes vs. no)	1.543 (1.222-1.949)	**0.000**	1.374 (1.084–1.742)	**0.009**
Tumor differentiation	1.158 (0.926-1.450)	0.199		
TNM (I–II vs. III–IV)	1.163 (0.945-1.430)	0.154		
LRG1 expression (low vs. high)	1.386 (1.124-1.708)	**0.002**	1.280 (1.037–1.581)	**0.022**

AFP, a-fetoprotein; HBsAg, hepatitis B surface antigen; HR, hazard ratio; CI, confidence interval.

### Effect of LRG1 on cell proliferation and migration

**Figure 6:**
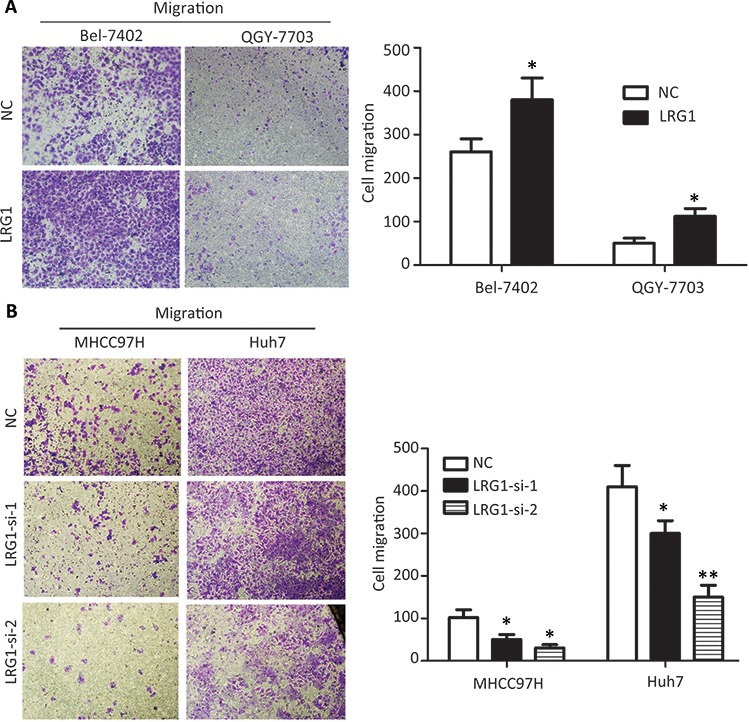
LRG1 promotes HCC cell migration Transwell assays demonstrated that LRG1 was capable of increasing cell migration abilities in HCC cells. **A.** Cells with or without LRG1 overexpression were seeded into transwells and cultured with medium without FBS for 48 h. Migrated cells were stained with 0.05% crystal violet and counted under microscope. **B.** Migration assays were repeated using cells with or without LRG1 knockdown. Data were representative of three independent experiments. Values were expressed as mean ± SEM (*P < 0.05, **P < 0.01).

The effect of LRG1 on cell mobility ability was examined by transwell migration assay. The results revealed that exogenous overexpression of LRG1 significantly promoted the migratory potential in both Bel-7402 and QGY-7703 cells (Figure 6A and Supplementary Figure S2A), whereas knockdown of LRG1 dramatically reduce the migrated cells in MHCC97H and Huh7 cells (Figure 6B and Supplementary Figure S2B). MTT and colony formation assays were conducted to determine the effect of LRG1 on cell proliferation. Data showed no significant change of cell growth between cell with or with LRG1 overexpression and knockdown (Supplementary Figure S3).

## DISCUSSION

LRG1 is one member of leucine-rich family and is involved in protein-protein interactions, signal transduction, and cell adhesion [19]. Current literatures show that LRG1 is closely associated with cancer metastasis and poor prognosis, largely due to its effects on promoting cell invasion, angiogenesis, and migration. Zhong et al. reported that LRG1 knockdown by RNA interference inhibited cell growth and promoted apoptosis in glioblastoma *in vitro* and *in vivo* [20]. Lynch et al. demonstrated that LRG1, regulated by a potent suppressor miR-335, was involved in cell migration, invasion and metastasis [21]. In our study, LRG1 expression was frequently higher in HCC patients with larger size tumor and advanced stage, which indicate that LRG1 might be capable of interfering with the development of HCC.

Deregulation of LRG1 protein was found in human cancers. Liu et al. reported that LRG1 was overexpressed in both blood and tumor sections in non-small cell lung cancer (NSCLC) [12]. Li and colleagues provided evidence that LRG1 was increased in urinary exosome of NSCLC patients [22]. Sandanayake et al. found that serum LRG1 was increased in patients with biliary tract cancer, compared with benign disease and healthy controls [23]. In HCC, increased expression of LRG1 in the serum of patients with AFP-negative HBV-related HCC was noted [24]. Zhang et al. showed that LRG1 was decreased in HCC and suppressed cell migration [25]. However, according to our data, LRG1 expression at both mRNA and protein levels were up-regulated in HCC cell lines and tissues. Elevated expression of LRG1 was frequently accompanied with worse malignant phenomenon, such as larger tumor size, more advanced tumor stage, poorer tumor differentiation and more vascular invasion in a large cohort of 777 HCC cases (In Zhang's study, only 51 HCC samples were collected). Furthermore, our *in vitro* data demonstrated that overexpression of LRG1 in HCC cells resulted in enhanced ability of cell migration, which was supported by that HCC cells with high migration capability (Huh7 and MHCC97H) expressed higher level of LRG1 in both two studies. Our data was in line with the other studies indicating that increased LRG1 expression was presented in ovarian cancer [11], non-small cell lung cancer [12], gastric cancer [13], pancreatic cancer [14] and leukemia [15]. Collectively, we consider our data are more representative to show the expression of LRG1 in HCC.

The prognostic implication of LRG1 was rarely studied. Wen et al. reported that high LRG1 expression was connected with favorable prognosis in endometrial carcinoma [18]. Wu et al. showed that serum LRG1 was increased in late-staged patients with epithelial ovarian cancer [26]. In this study, LRG1 was identified as an independent factor for overall and disease-free survival in a large cohort of 777 patients with HCC. Patients with high LRG1 expression usually lived a shorter life. These data suggest that LRG1 is of clinical implication in predicting outcomes of cancer patients.

Several studies showed that LRG1 achieved its part biological function by regulating TGF-β signaling pathway [10, 27, 28], which plays an important role in the development of tumor [29]. Vogt et al. demonstrated that LRG1 was involved in the regulation of polar tip growth by affecting PKC/MAK1 pathway activities [30]. Cummings et al. reported that LRG1 was able to bind to and inhibit cytochrome *c* which is an essential activator of cell apoptosis [31]. These data suggest that LRG1 might exert functions towards tumor growth. In our study, LRG1 overexpression resulted in the enhancement of HCC cell mobility ability.

In summary, our data reveal that LRG1 was frequently up-regulated in HCC and promoted HCC cells mobility ability. Increase of LRG1 expression was significantly correlated with tumor size, tumor differentiation, TNM stage and vascular invasion, suggesting that LRG1 might play a role in HCC progression. High LRG1 expression unfavorably impacted the survival of HCC patients and served as an independent factor for worse outcomes. Collectively, our data suggest LRG1 is a promising biomarker for prognosis of patients with HCC.

## MATERIALS AND METHODS

### Patients, tissue specimens and follow-up

A total of 777 paraffin-embedded HCC specimens between January 2000 and December 2010 were obtained from the archives of the Department of Pathology of the Sun Yat-sen University Cancer Center. None of the patients received any chemotherapy or radiotherapy prior to the surgery. We randomly divided these cases into a training cohort (*n* = 474, 61.0%) and an independent validation cohort (*n* = 303, 39.0%). The follow-up period was defined as the interval from the date of surgery to the date of death or the last follow-up. This study has been approved by the Institute Research Medical Ethics Committee of SYSUCC.

### Tissue microarray (TMA) construction and Immunohistochemistry

The TMA slides included 777 HCC and adjacent nontumorous liver tissues. Using a tissue array instrument (Minicore excilone, Minicore, British), each tissue core with a diameter of 0.6 mm was punched from the marked areas and re-embedded. All specimens were fixed at 4% paraformaldehyde in 0.1 M phosphate buffer for 24 h and embedded in paraffin wax. Then the paraffin-embedded HCC sections were sliced into 4 μm and mounted onto glass slides. After dewaxed, the slides were treated by 3% hydrogen peroxide in methanol and blocked by a biotin-blocking kit (DAKO, Germany). After blocking, the slides were incubated with LRG1 antibody (1:1000, Sigma, Cambridge, England) overnight in a moist chamber at 4°C. After washed in PBS for three times, the slides were incubated with biotinylated goat anti-rabbit antibodies for 1 h. Then the slides were stained with the DAKO Liquid 3,′3-diaminobenzidine tetrahydrochloride (DAB). Finally, the slides were counter stained with Mayer's hematoxylin and observed under microscope.

The protein level was determined by Semi-quantitative immunohistochemistry (IHC) detection. The positively-stained was scored as follow: “0” (less than 5% positively-stained cells), “1” (6–24% of positively-stained cells), “2” (25–49% of positively-stained cells), “3” (50–74% of positively-stained cells) and “4” (75%–100% of positively-stained cells). Intensity was scored was according to the standard: “0” (negative staining); “1” (weak staining); “2” (moderate staining) and “3” (strong staining). The final score was served by multiplying the percentage score by the staining intensity score. The scores were independently decided by two pathologists (Dr. Jing-Ping Yun and Dr. Min Li). The median IHC score was chosen as the cut-off value for defining high and low expression.

### Western blot

Total proteins were extracted and separated by 10% SEMS-PAGE and then transferred onto PVDF membrane (Millipore, Bedford, MA). Equal amounts of protein (30 μg) were resolved by SDS-PAGE and then electrophoretically transferred onto PVDF membranes. After blocked in 5% non-fat milk 1 h at room temperature, the membranes were incubated with appropriately diluted primary antibodies overnight at 4°C. After washed thrice with TBST, The blotted membranes were incubated with anti-LRG1 (1:1000, Sigma, Cambridge, England). The membranes were incubated with HRP-conjugated secondary antibody at 1:20000 dilutions for 1 h at room temperature. The membranes were visualized by the enhanced Phototope TM-HRP Detection Kit and exposed to Kodak medical X-ray processor (Carestream Health, USA). Anti-GAPDH (1:1000, Santa Cruz, CA, USA) was used as a loading control.

### Quantitative real-time RT-PCR (qRT-PCR)

Total RNA was extracted from clinical samples and cultured cells using Trizol reagent (BIOO Scientific Co., USA) following manufacture instruction. The reverse transcription with random primers was done by M-MLV Reverse Transcriptase (Promega Inc., USA) according to the manufacturer's instructions. SYBR green-based real-time PCR as carried out to measure levels of the corresponding *LRG1* and 18S by the Strata gene Mx3000P Real-time PCR system. Primers were designed as follows: *LRG1*, forward: 5′-CCATCTCCTGTCAACCACCT-3′ and reverse: 5′-GTTTCGGGTTAGATCCAGCA-3′; 18S, forward: 5′-TGAGAAACGGCTACCACATCC-3′ and reverse: 5′-ACCAGACTTGCCCTCCAATG-3′. The qRT-PCR reactions were done 95°C for 10 min for initial denaturation, and then 95°C for 30 seconds, 60°C for 30 seconds, 72°C for 30 seconds and a final extension of 10 min for 40 cycles. SDS 2.3 software (Applied Biosystems) was used to quantify and analyze the relative mRNA levels. Relative quantification of LRG1 mRNA was performed using the 2^−ΔCt^ method. The experiments were done at least thrice independently and all samples were in triplicate.

### Migration assay

For the migration assay, 2–4 × 10^4^ cells in serum-free medium were plated in the upper compartment of a transwell chamber (8-μm pore size, Millepore, USA). After incubation for 24–48 hours, the migrated cells on the lower membrane were counted after staining with 0.1% crystal violet and 20% methanol. The experiment was performed in triplicate and repeated three times.

### MTT and colony formation assays

After transfection, cells were seeded in 96-well plates (3 × 10^4^ cells/ml) with 100 ul medium in each well and cultured for 5 days. MTT assay was performed by adding 20 ul of MTT (5 mg/ml, AMRESCO, Solon, OH, USA) for 4 h at 37°C. Then, the formazan crystals were dissolved in DMSO (150 μl/well). The absorbance at 490 nm of each sample was measured using a multilabel plate reader (PerkinElmer). For the colony formation assay, 500 cells were seeded into 6-well plates and incubated at 37°C in a humidified atmosphere containing 5% CO2 in air for 10–14 days. Colonies were fixed with methanol, stained with 0.1% crystal violet and counted.

### Plasmid construction and transfection

The recombinant plasmids pcDNA 3.1/hygro(+) vector and pcDNA 3.1/hygro(+)-LRG1 were confirmed by sequencing. We constructed the plasmids into QGY-7703 and Bel-7402 cells by Lipofectamine™ 2000 (Invitrogen; Carlsbad, CA, USA) transduction. After an antibiotic selection with 800 μg/ml G418 (Clontech, CA), the G418-resistant clone was isolated and expanded into cell lines and tested the expression of LRG1 by western blot.

### RNA interference

siRNA duplexes targeted LRG1 (siRNA#1: forward 5′-CAUGCUGGACCUCUCCAAUTT-3′, reverse 5′-AUU GGAGAGGUCCAGCAUGTT-3′; siRNA#2: forward: 5′-CCUGAGCGACCUCUAUCGUTT-3′, reverse: 5′-AC GAUAGAGGUCGCUCAGGTT-3′;) and negative control (NC) siRNA duplex (forward: 5′-UUCUCCGAA CGUGUCACGUTT-3′; reverse: 5′-ACGUGACACGUUC GGAGAATT-3′) were chemically synthesized by Shanghai GenePharma Co. Ltd (Shanghai, China). Transfection was performed using the Lipofectamine™ RNAiMAX (Invitrogen; Carlsbad, CA, USA) according to the manufacturer's instructions.

### Statistical analysis

Statistical analysis was performed using the SPSS (version 16.0, Chicago, IL). The data for LRG1 expression was analyzed by using the Student's *t*-test. Pearson's χ^2^ test or Fisher's exact test was chosen for examining the correlations between LRG1 expression level and the clinical and pathological variables. Survival curves were carried out by the Kaplan-Meier method (log-rank test). Multivariate Cox proportional hazards regression model was conducted to evaluate the independence of LRG1 in prognosis. Differences were considered significant for *P* value less than 0.05.

## SUPPLEMENTARY FIGURES AND TABLES


