# Supplementary material for: LRG1 expression indicates unfavorable clinical outcome in hepatocellular carcinoma
Source: Oncotarget. 2015 Oct 19;6(39):42118–29. doi: 10.18632/oncotarget.5967 (PMC4747214; doi:10.18632/oncotarget.5967)
Supplement: Supplementary file 1 [file oncotarget-06-42118-s001.pdf]

SUPPLEMENTARY FIGURES AND TABLES

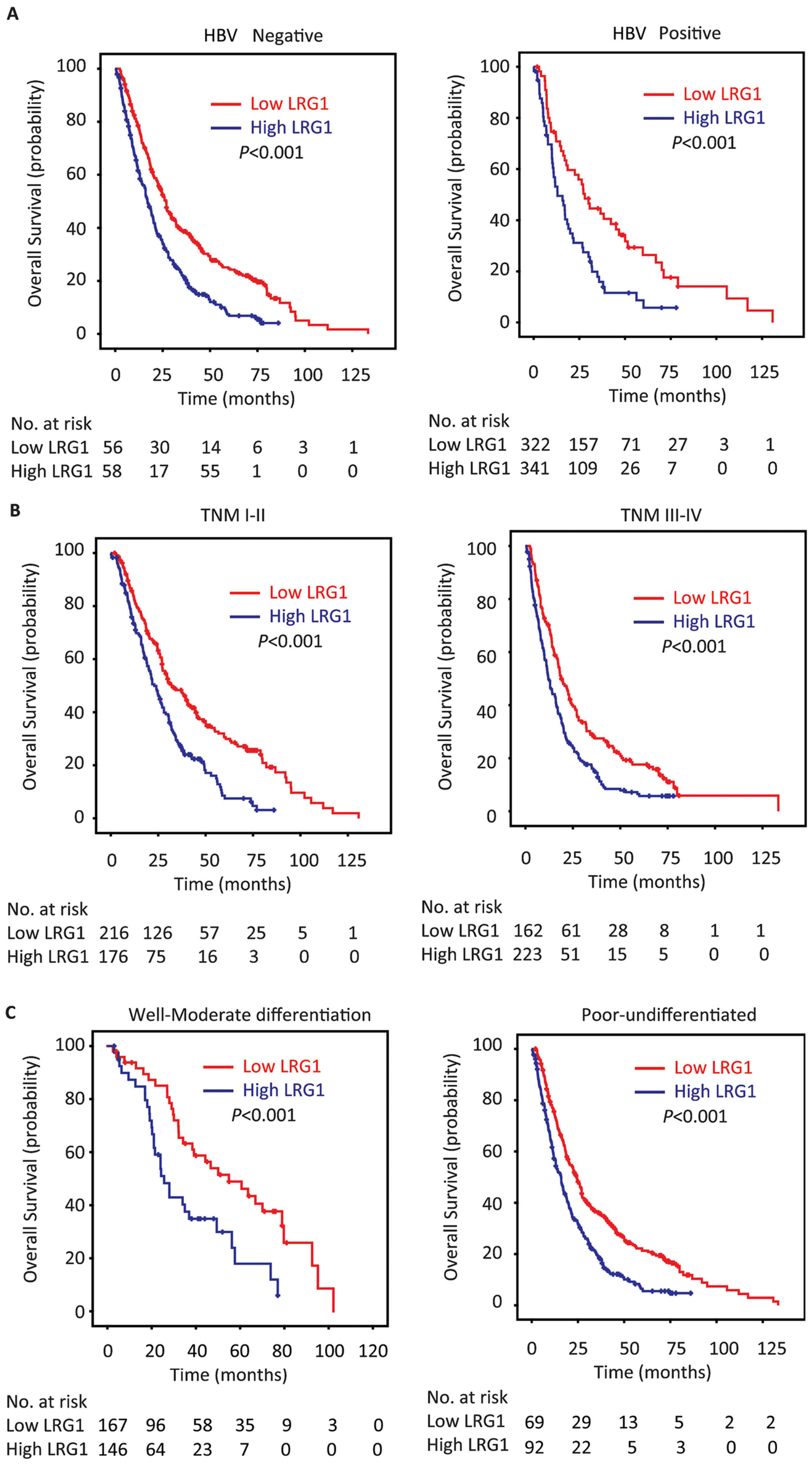

Supplementary Figure S1: Stratified survival analyses in another 3 subgroups (HBV, negative vs. positive, TNM, I–II vs. III–IV, tumor differentiation, well-moderate vs. poor-undifferentiated) for overall survival were shown.

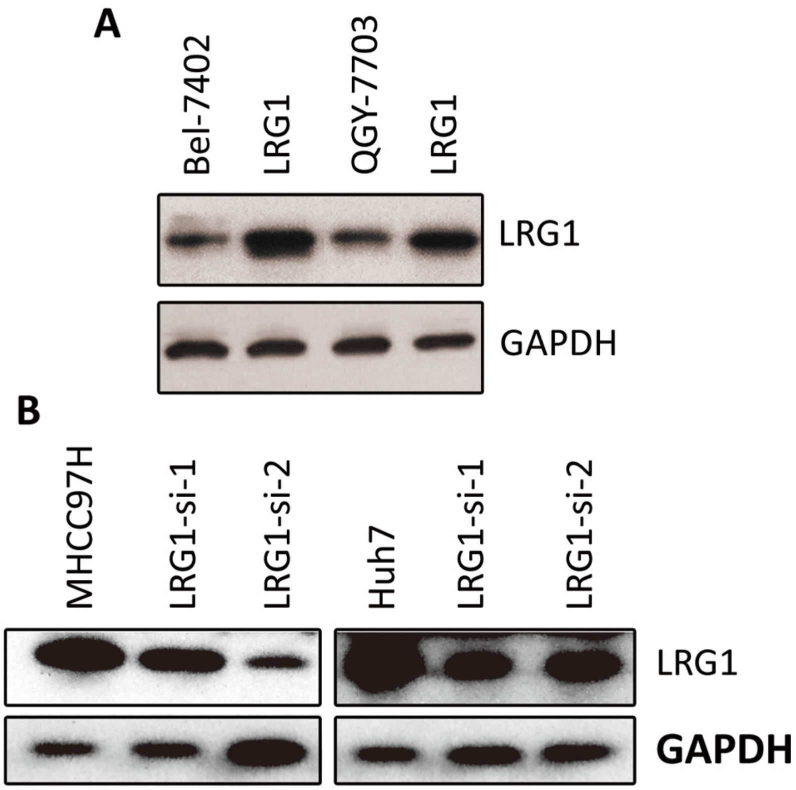

Supplementary Figure S2: Western blot was performed to examined the overexpression A. and knockdown B. of LRG1 in HCC cells.

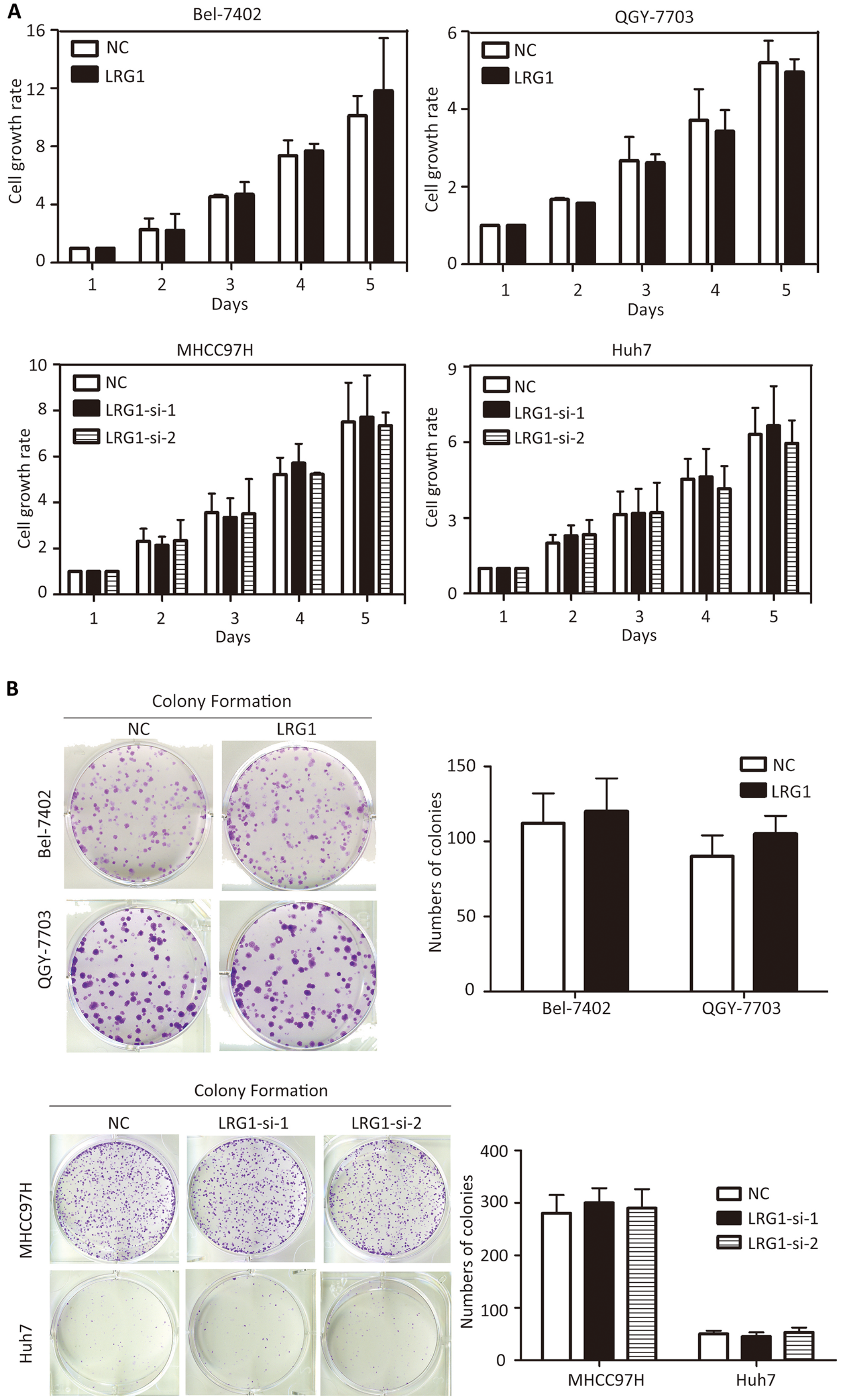

Supplementary Figure S3: Cell proliferation was determined by MTT A. and colony formation B. in cells with LRG1 overexpression or silence. Data were representative of three independent experiments. Values were expressed as mean ± SEM.

Supplementary Table S1: Correlation of clinicopathological parameters and LRG1 expression in the training and validation cohort

Variable	Training cohort				Validation cohort			
	All cases	Low expression	High expression	*P* value[a]	All cases	Low expression	High expression	*P* value[a]
Age (years)[b]				0.994				0.228
<49	217	108 (49.8%)	109 (50.2%)		151	76 (50.3%)	75 (49.7%)	
≥49	257	128 (49.8%)	129 (50.2%)		152	66 (43.4%)	86 (56.6%)	
Gender				0.168				0.268
Male	436	213 (48.9%)	223 (51.1%)		263	120 (45.6%)	143 (54.4%)	
Female	38	23 (60.5%)	15 (39.5%)		40	22 (55.0%)	18 (45.0%)	
HBsAg				0.410				0.412
Positive	406	199 (49.0%)	207 (51.0%)		257	123 (47.9%)	134 (52.1%)	
Negative	68	37 (54.4%)	31 (45.6%)		46	19 (41.3%)	27 (58.7%)	
AFP (ng/ml)				0.232				0.306
<20	127	69 (54.3%)	58 (45.7%)		102	52 (51.0%)	50 (49.0%)	
≥20	347	167 (48.1%)	180 (51.9%)		201	90 (44.8%)	111 (55.2%)	
Cirrhosis				0.963				0.139
Yes	376	187 (49.7%)	189 (50.3%)		243	119 (49.0%)	124 (51.0%)	
No	98	49 (50.0%)	49 (50.0%)		60	23 (38.3%)	37 (61.7%)	
Tumor size (cm)				**0.037**				**0.035**
<5	136	78 (57.4%)	58 (42.6%)		99	55 (55.6%)	44 (44.4%)	
≥5	338	158 (46.7%)	180 (53.3%)		204	87 (42.6%)	117 (57.4%)	
Tumor multiplicity				0.185				0.143
Single	281	147 (52.3%)	134 (47.7%)		183	92 (50.3%)	91 (49.7%)	
Multiple	193	89 (46.1%)	104 (53.9%)		120	50 (41.7%)	70 (58.3%)	
Differentiation				**0.030**				0.128
Well-Moderate	313	167 (53.4%)	146 (46.6%)		220	109 (49.5%)	111 (50.5%)	
Poor-undifferentiated	161	69 (42.9%)	92 (57.1%)		83	33 (39.8%)	50 (60.2%)	
TNM				**0.008**				**0.012**
I–II	234	131 (56.0%)	103 (44.0%)		158	85 (53.8%)	73 (46.2%)	
III–IV	240	105 (43.8%)	135 (56.3%)		145	57 (39.3%)	88 (60.7%)	
Vascular invasion				0.419				**0.006**
Yes	114	53 (46.5%)	61 (53.5%)		73	24 (32.9%)	49 (67.1%)	
No	360	183 (50.8%)	177 (49.2%)		230	118 (51.3%)	112 (48.7%)	
Involucrum				0.457				0.161
Complete	163	85 (52.1%)	78 (47.9%)		105	55 (52.4%)	50 (47.6%)	
Incomplete	311	151 (48.6%)	160 (51.4%)		198	87 (43.9%)	111 (56.1%)	

[a]Chi-square test;
[b]Median age; AFP, alpha-fetoprotein; HBsAg, hepatitis B surface antigen.

Supplementary Table S2: Univariate and multivariate analyses of clinicopathological and LRG1 expression for overall survival in the training and validation cohort

Variables	Univariate analysis		Multivariate analysis	
	HR (95% CI)	*P* value	HR (95% CI)	*P* value
**Training cohort (*n* = 474)**				
Age (<49 vs. ≥49 years)	1.074 (0.879–1.311)	0.484		
Gender (female vs. male)	1.009 (0.695–1.465)	0.961		
HBV (positive vs. negative)	0.919 (0.696–1.214)	0.554		
Tumor size (<5 vs. ≥5 cm)	1.907 (1.504–2.416)	**0.000**	1.706 (1.325–2.196)	0.814
Tumor multiplicity (single vs. multiple)	1.245 (1.018–1.522)	**0.033**	1.043 (0.813–1.336)	0.740
Invonucrum (absent vs. present)	1.145 (0.933–1.405)	0.193		
Liver cirrhosis (yes vs. no)	1.179 (0.911–1.525)	0.209		
AFP (<20 vs. ≥20 ng/mL)	1.770 (1.393–2.246)	**0.000**	1.566 (1.228–1.996)	**0.000**
Vascular invasion (yes vs. no)	1.854 (1.471–2.335)	**0.000**	1.555 (1.198–2.018)	**0.001**
Tumor differentiation	1.314 (1.065–1.621)	**0.011**	1.134 (0.911–1.410)	0.259
TNM (I–II vs. III–IV)	1.539 (1.260–1.879)	**0.000**	0.986 (0.745–1.303)	0.918
LRG1 expression (low vs. high)	1.787 (1.456–2.192)	**0.000**	1.699 (1.383–2.087)	**0.000**
**Validation cohort (*n* = 303)**				
Age (<49 vs. ≥49 years)	0.963 (0.746–1.242)	0.773		
Gender (female vs. male)	0.864 (0.591–1.260)	0.448		
HBV (positive vs. negative)	1.071 (0.747–1.534)	0.701		
Tumor size (<5 vs. ≥5 cm)	2.023 (1.507–2.715)	**0.000**	1.515 (1.097–2.091)	**0.011**
Tumor multiplicity (single vs. multiple)	1.400 (1.082–1.809)	**0.010**	0.943 (0.679–1.308)	0.723
Invonucrum (absent vs. present)	1.083 (0.832–1.409)	0.551		
Liver cirrhosis (yes vs. no)	0.908 (0.655–1.257)	0.562		
AFP (<20 vs. ≥20 ng/mL)	2.275 (1.693–3.057)	**0.000**	2.089 (1.545–2.824)	**0.000**
Vascular invasion (yes vs. no)	1.967 (1.469–2.633)	**0.000**	1.305 (0.935–1.820)	0.116
Tumor differentiation	1.448 (1.095–1.914)	**0.009**	1.107 (0.829–1.476)	0.488
TNM (I–II vs. III–IV)	2.206 (1.564–2.623)	**0.000**	1.558 (1.070–2.266)	**0.020**
LRG1 expression (low vs. high)	1.711 (1.318–2.220)	**0.000**	1.421 (1.080–1.867)	**0.011**

AFP, α-fetoprotein; HBsAg, hepatitis B surface antigen; HR, hazard ratio; CI, confidence interval.

Supplementary Table S3: Univariate and multivariate analyses of clinicopathological and LRG1 expression for disease-free survival in the training and validation cohort

Variables	Univariate analysis		Multivariate analysis	
	HR (95% CI)	*P* value	HR (95% CI)	*P* value
**Training cohort (*n* = 474)**				
Age (<49 vs. ≥49 years)	1.168 (0.898–1.519)	0.246		
Gender (female vs. male)	0.901 (0.549–1.477)	0.679		
HBV (positive vs. negative)	1.024 (0.700–1.496)	0.904		
Tumor size (<5 vs. ≥5 cm)	1.379 (1.031–1.845)	**0.030**	1.267 (0.944–1.700)	0.116
Tumor multiplicity (single vs. multiple)	0.967 (0.741–1.264)	0.808		
Invonucrum (absent vs. present)	1.116 (0.853–1.459)	0.422		
Liver cirrhosis (yes vs. no)	1.335 (0.949–1.878)	0.098		
AFP (<20 vs. ≥20 ng/mL)	1.604 (1.181–2.178)	**0.002**	1.494 (1.096–2.037)	**0.011**
Vascular invasion (yes vs. no)	1.467 (1.088–1.976)	**0.012**	1.318 (0.973–1.785)	0.074
Tumor differentiation	1.001 (0.754–1.327)	0.997		
TNM (I–II vs. III–IV)	1.082 (0.834–1.404)	0.554		
LRG1 expression (low vs. high)	1.359 (1.044–1.769)	**0.023**	1.303 (1.001–1.698)	**0.049**
**Validation cohort (*n* = 303)**				
Age (<49 vs. ≥49 years)	0.853 (0.607–1.198)	0.359		
Gender (female vs. male)	1.438 (0.909–2.275)	0.120		
HBV (positive vs. negative)	1.236 (0.752–2.033)	0.403		
Tumor size (<5 vs. ≥5 cm)	1.725 (1.173–2.536)	**0.006**	1.533 (1.032–2.277)	**0.034**
Tumor multiplicity (single vs. multiple)	1.334 (0.946–1.880)	0.100		
Invonucrum (absent vs. present)	1.324 (0.936–1.873)	0.112		
Liver cirrhosis (yes vs. no)	0.763 (0.510–1.140)	0.187		
AFP (<20 vs. ≥20 ng/mL)	1.583 (1.094–2.291)	**0.015**	1.434 (0.984–2.089)	0.061
Vascular invasion (yes vs. no)	1.666 (1.143–2.428)	**0.008**	1.483 (1.008–2.180)	**0.045**
Tumor differentiation	1.493 (1.030–2.162)	**0.034**	1.360 (0.935–1.979)	0.108
TNM (I–II vs. III–IV)	1.303 (0.926–1.832)	0.128		
LRG1 expression (low vs. high)	1.436 (1.017–2.027)	**0.040**	1.206 (0.844–1.724)	0.303

AFP, a-fetoprotein; HBsAg, hepatitis B surface antigen; HR, hazard ratio; CI, confidence interval.
